# Structure and mechanism of an antibiotics-synthesizing 3-hydroxykynurenine C-methyltransferase

**DOI:** 10.1038/srep10100

**Published:** 2015-05-11

**Authors:** Sheng-Chia Chen, Chi-Hung Huang, Shu-Jung Lai, Jai-Shin Liu, Pin-Kuei Fu, Shih-Ting Tseng, Chia Shin Yang, Mei-Chin Lai, Tzu-Ping Ko, Yeh Chen

**Affiliations:** 1 Department of Biotechnology, Hungkuang University, Taichung, Taiwan; 2 Taiwan Advance Biopharm (TABP), Inc., Xizhi City, New Taipei City, Taiwan; 3 Department of Life Sciences, National Chung Hsing University, Taichung, Taiwan; 4 Institute of Bioinformatics and Structural Biology, National Tsing Hua University, Hsinchu, Taiwan; 5 Division of Critical Care & Respiratory Therapy, Department of Internal Medicine, Taichung Veterans General Hospital, Taichung, Taiwan; 6 Department of Applied Chemistry, National Chi Nan University, Nantou County, Taiwan; 7 Department of Food and Nutrition, Providence University, Taichung, Taiwan; 8 Department of Endocrinology and Metabolism, Kuang Tien General Hospital, Taiwan; 9 Institute of Biological Chemistry, Academia Sinica, Taipei, Taiwan

## Abstract

*Streptosporangium sibiricum* SibL catalyzes the methyl transfer from S-adenosylmethionine (SAM) to 3-hydroxykynurenine (3-HK) to produce S-adenosylhomocysteine (SAH) and 3-hydroxy-4-methyl-kynurenine for sibiromycin biosynthesis. Here, we present the crystal structures of apo-form *Ss*-SibL, *Ss*-SibL/SAH binary complex and *Ss*-SibL/SAH/3-HK ternary complex. *Ss*-SibL is a homodimer. Each subunit comprises a helical N-terminal domain and a Rossmann-fold C-terminal domain. SAM (or SAH) binding alone results in domain movements, suggesting a two-step catalytic cycle. Analyses of the enzyme-ligand interactions and further mutant studies support a mechanism in which Tyr134 serves as the principal base in the transferase reaction of methyl group from SAM to 3-HK.

The *Streptosporangium sibiricum* SibL (*Ss*-SibL) protein belongs to the S-adenosylmethionine-dependent methyltransferase (SAM-dependent MTase) family. It transfers a methyl group from S-adenosyl-l-methionine (SAM) to 3-hydroxykynurenine (3-HK) to produce S-adenosyl-l-homocysteine (SAH) and 3-hydroxy 4-methyl-kynurenine (3-H4MK) (Fig. 1). 3-H4MK is the key intermediate needed to synthesize pyrrolo-[1, 4] benzodiazepine (PBD) antitumor antibiotics, including sibiromycin and anthramycin, in *Streptomycetes*^1^,^2^,^3^. Sibiromycin is a potent natural antibiotic produced by *S. sibiricum* and has been found to inhibit the growth of many bacteria, including *Bacillus mycoides, B. subtilis,* and *Staphylococcus aureus*^4^. It also exhibits very high DNA binding affinity and good specificity as a DNA-alkylating agent, and has been investigated with regard to its anti-tumor properties^5^,^6^,^7^.

Sibiromycin, anthramycin and tomaymycin are PBD derivatives with a pharmacophore consisting of a tricyclic moiety (anthranilate, diazepine, and hydropyrrole) that is biosynthesized by combining l-tryptophan, l-tyrosine, and l-methionine^2^,^8^. The precise sequence of reactions that turn out the PBD antibiotics has been characterized biochemically. It depends on the specific moieties of individual antibiotic. Sibiromycin and anthramycin are both derived from 3-H4MK with a common C8-methyl group (Supplementary Fig. S1), where tomaymycin has a hydroxyl group. Different tricyclic ring substitutions of PBDs result in distinct biological activities. For example, having a C9-hydroxyl group in sibiromycin and anthramycin can cause cardiotoxicity^1^,^3^,^9^,^10^; O-glycosylation of sibirosamine at C7 significantly enhances the DNA binding affinity of sibiromycin^3^,^5^,^11^,^12^,^13^. All these compounds are capable of forming covalent DNA-antibiotic adducts and are potent antitumor drugs. Notably, sibiromycin effectively competes with the other two antibiotics and also has a faster reaction rate^14^.

As the second most widely used enzyme substrate (after ATP), SAM is not only a source of methyl group for transmethylation via S_N_2 mechanism^15^, but it also provides homocysteine for transsulfuration and supplies aminopropyl group for aminopropylation^16^. Regarding transmethylation, about 120 members of SAM-dependent MTs have been classified (EC 2.1.1.X) based on their substrate specificity and on the targeted atom for methylation^17^. O-directed MTs are the most abundant (54%), whereas C-directed MTs constitute only 18%. There are five known families of SAM-dependent MT (class I-V) with distinct protein folds of the catalytic domain^18^. The core structure of class I MT is a Rossmann-like α/β fold, class II contain a TIM-barrel β/α core domain, class III has a tetrapyrrole methylase α/β fold structure, while class IV and class V include the SPOUT α/β structure and an all-β SET domain^18^.

Because class I MTs show the highest diversity, they are subclassified according to the substrate specificity for small molecule, DNA, RNA, lipid and protein, plus several other uncharacterized enzymes^17^. Although these SAM-dependent MTs share low sequence identity, they all contain a similar Rossmann-like core domain that comprises alternating β strands (β1-β7) flanked by α helices, with a highly conserved SAM binding motif (GxG motif) located in strand β1^17^. On the other hand, a recent study of the *Saccharomyces cerevisiae* methyltransferome identified four novel classes of MT (class VI to class IX) with core structures of transmembrane all-α, DNA/RNA-binding 3-helical bundle all-α, SSo0622-like α/β and thymidylate synthetase α/β folds, respectively^19^.

Evidence suggests that the family of natural product methyltransferases (NPMTs) is a result of concomitant gene fusions, which led to neofunctionalization of substrate recognition by new domains^20^,^21^,^22^,^23^. NPMTs usually methylate their substrates through an SN_2_-like nucleophilic substitution reaction that is categorized by three distinct mechanisms: 1) proximity and desolvation effects (PD), 2) general acid-base catalysis and 3) metal-dependent mechanisms^21^. To elucidate the catalytic mechanism, structural and biochemical approaches have a strong contribution to identify whether pivotal residues or metal ions exist or not. For example, DnrK is considered to use PD mechanism because the mutagenesis of Tyr142, a key residue nearby the catalytic center, did not dramatically reduce the enzyme activity^24^. As for general acid-base catalysis, many methyltransferases have been structurally and biochemically characterized to use a general base in active site for catalysis^21^. NovP and MycE utilize a divalent ion to coordinate substrate and a nearby residue abstracts proton form methyl acceptor^25^,^26^. Therefore, understanding the roles of crucial residues near the active center from structure perspective seems to be indispensable in NPMTs.

Structure prediction programs such as Phyre^27^ revealed *Ss*-SibL shares high structural similarity to the class I O-MTs in the structural fold, despite low sequence identity (<30%). However, biochemical experiments showed its kinship to C-MT in that it transfers a methyl group to the C4 position^7^,^28^. According to the phylogenetic analysis among O-MT and C-MT, Ss-SibL was classified into C-MT and supposed to be similar to AcmI and AcmL (with ~55% identity) in structure and mechanism^29^. To date, knowledge about the biochemical characteristics of these enzymes is still limited.

To investigate the underlying mechanism of *Ss*-SibL catalysis and substrate recognition, we determined its crystal structure by using single-wavelength anomalous dispersion (SAD) method. The unbound *Ss*-SibL, a binary complex with SAH and a ternary complex with both SAH and 3-HK provide the first picture of direct interactions between the protein and its cofactor/substrate in this family. A comparison of the unbound and complex structures suggests that SAH binding triggers domain closure to form a pocket favorable for 3-HK binding. Identification of crucial amino acids for 3-HK binding suggests that Tyr99, Tyr134 and Tyr295 may be involved in catalysis. The pivotal role of Tyr134 is further confirmed by mutagenesis. These results provide new insights into the mechanism of the methyl transferase.

## Results

### Overall structure

The structure of selenomethionine (SeMet)-substituted *Ss*-SibL in complex with substrate and cofactor was first determined using single-wavelength anomalous dispersion (SAD). The apo form structure was then solved by molecular replacement. Two monomers are tightly intertwined head-to-head, with ~23% total solvent accessible area buried in the dimer interface. Dimer formation is crucial to the stability of SibL^24^,^30^,^31^. Each subunit has two domains. The N-terminal domain (residues 4–139 and 282–299) is composed of several helices and a β-ribbon. These helices bundle with those of the other subunit mainly via hydrophobic interactions and promote dimer formation. The C-terminal domain is composed of a parallel β-sheet surrounded by α-helices, exhibiting a typical *Rossmann* fold that contains the nucleotide binding site for accommodating the cofactor^17^,^32^. Between the two domains, helix α11 is considered to balance tension upon conformational change. Here a broad pocket is formed, which shows an opening on the protein surface, most likely to accommodate the substrate and the cofactor. The final model was refined to an R-value of 21.1% (R_free_ = 26.5%), and the crystallographic statistics are given in Table 1^33^.

**Figure 2:**
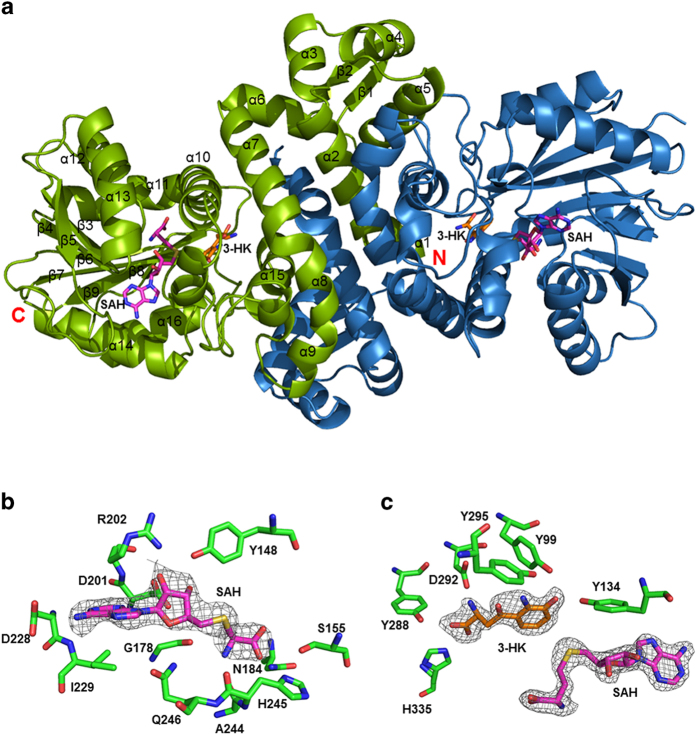
Structure of the *Ss*-SibL protein. (**a**) Ribbon representation of the dimeric Ss-SibL structure is viewed down its 2-fold axis. Subunits A and B are shown in green and blue, respectively. The SAH and 3-HK are shown as magenta and orange stick models. (**b**) and (**c**) The composite omit maps for the ligands in the binary and ternary complex crystals are contoured at the 1.5 σ level and drawn as grey mesh presentations. Each subunit of the bound *Ss*-SibL includes both SAH and 3-HK in the cleft. SAH is depicted as sticks with the carbon, oxygen, nitrogen, and sulfur atoms in magenta, red, blue, and yellow, whereas 3-HK is illustrated with the carbon, oxygen, and nitrogen in orange, red, and blue, respectively.

**Figure 3:**
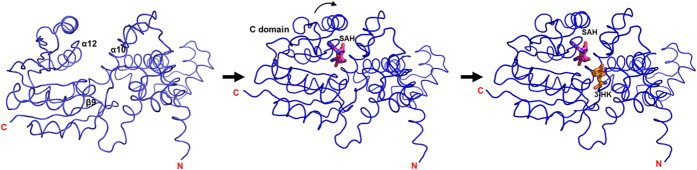
The conformational change of *Ss*-SibL. Structures of *Ss*-SibL reveal that conformational change of the C-terminal domain triggered by SAH binding, followed by 3-HK binding that does not cause any major structural change.

In the presence of SAM or SAH and 3-HK, the binary *Ss-*SibL/SAH and the ternary *Ss-*SibL/SAH/3HK complex crystals diffracted to higher resolution of 2.6 Å and 2.1 Å. The latter structure is shown in Fig. 2a. Although the *Ss*-SibL/SAH crystals were obtained by using SAM, the methyl group lacked corresponding densities and the ligand could only be modeled as the demethylated product (Fig. 2b). Possibility could be either (1) SAM lost its methyl group spontaneously during crystallization; or (2) the methyl group of SAM was removed *in situ*^34^,^35^. In the ternary complex crystal, both ligands SAH and 3-HK fit well with the extra electron densities found inside the active-site pocket (Fig. 2c). Although d-/l-3-HK was used in crystallization, the electron densities clearly indicate that the bound substrate is l-3-HK, consistent with the lower Km value for l-3-HK (210 μM) than d-3-HK (311 μM)^7^. Each ligand-bound subunit of *Ss*-SibL showed a similar and robust architecture in its peptide backbone, whereas a 2.1 Å r.m.s.d. in the 294 Cα positions was seen in the unbound structure, indicating a considerable conformational rearrangement upon ligand binding (Fig. 3).

Superposition of apo form and binary complex structures indicates a rotation of the C-terminal domain of approximately 22° toward the N-terminal domain^36^. Close to the bound SAH a highly conserved DXGGGXA loop connects strand β3 to helix α12, which has been identified as the SAM-binding motif^31^. In the binary complex structure, several hydrogen bonds were found between SAH and the C-terminal domain, that provide a major force to strain the C-terminal domain into the closed conformation. These interactions with SAH cause the helix α10 backbone to shift ~2.4 Å closer to the β9 sheet. The concomitant flipping of helix α12 is the most significant structural change that occurs upon ligand binding, with approximately ~5.5 Å shift of the Cα atoms (Fig. 3). Interestingly, further binding of 3-HK does not result in significant conformational change in the protein backbone. Only minor differences in the side chains are seen when the binary and ternary complex structures are compared.

Based on these observations, a step-wise reaction of SibL can be proposed in which cofactor binding first brings the two domains close together to complete substrate-binding site formation. In the following step the substrate enters into this site for methyl transfer. Similar conformational changes have also been described in other methyltransferases^17^,^21^,^37^. For example, a comparison of the binary (with SAM) and the ternary (with SAM and ASE) complex structures of the human enzyme ASMT did not show major shifts in its backbone, indicating that cofactor binding is sufficient to induce the closed conformation and no further domain movement occurs upon substrate binding^38^. Therefore, SAM binding that triggers conformational changes seems to be a common mechanism in Ss-SibL and some class I O-MTs, which provides a thermodynamically favored environment for the substrate binding.

### The SAH/3-HK binding sites

**Figure 4:**
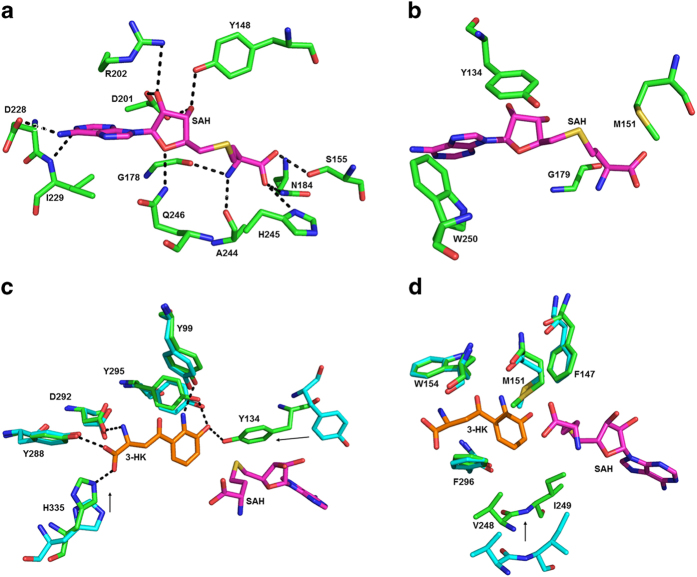
Ligand interactions in the active site. In (**a**) the SAH-interacting residues via hydrogen bonds are shown as stick models. The nitrogen, oxygen, and carbon atoms are colored blue, red, and green. The bound SAH is shown with magenta carbons. Black dashes represents the potential hydrogen bonds. The remaining SAH-interacting residues are shown in (**b**). The 3HK-interacting residues via hydrogen bonds are shown in (**c**) and the remaining residues in (**d**). For comparison, the residues from the unbound structure are also shown in (**c**) and (**d**), with cyan carbons.

The ternary complex crystal of *Ss-*SibL/SAH/3HK that we obtained by cocrystallization is the first example of a *Ss*-SibL in which both the cofactor and the substrate are stably bound to the active site. The cofactor SAH is accommodated deep in a pocket, which is formed by four loops, two strands and two helices. There are eleven direct hydrogen bonds between ten protein residues and the cofactor. Met151, Ser155, Gly178, Gly179, Asn184, Ala244 and His245 interact with the terminal amino acid moiety of SAH; Tyr134, Arg202, Asp228, Ile229 and Trp250 interact with the adenine ring; and Tyr148, Asp201, Arg202, and Gln246 interact with the ribose moiety, making five of the eleven hydrogen bonds (Figs. 4a,b). Identical binding mode is seen in the binary complex of *Ss-*SibL/SAH. These contacts, which are specific for SAH, appear to contribute to the major forces that not only recognize the cofactor but also induce the above mentioned conformational change.

Adjacent to the cofactor, the substrate 3-HK appear to be entirely buried in the active-site pocket, which is constituted mostly by α-helices from both N- and C-terminal domains. Six direct hydrogen bonds are formed between 3-HK and Tyr99, Tyr134, Tyr288, Asp292, Tyr295, and His335, which are located in helices α7, η3, α15 and strand β9 (Fig. 4c). Together these residues recognize the substrate and hold it in a specific orientation required for transmethylation. Besides, a number of other residues (Phe147, Met151, Trp154, Val248, Ile249 and Phe296) provide hydrophobic interactions to complementarily secure 3-HK in a nonpolar environment (Fig. 4d). The kynurenine ring is sandwiched between the Met151 and Phe296 side chains, while making a T-stacking with the latter.

When comparing the bound and unbound forms of *Ss-*SibL we observe that the peptide backbone from strand β6 to helix η5 is shifted by ~4.6 Å to constrict the active site upon substrate binding. The movement facilitates hydrogen bond formations of Asp292 and Tyr288 with the amino acyl moiety of 3-HK and thus plays an important role in stabilizing and recognizing the substrate. The β9 strand is also shifted slightly, bringing His335 into a position to form a hydrogen bond with the carboxyl group. Thus, the residues near the polar tail of 3-HK provide a major interactions for substrate selection. For example, 3-hydroxyanthranilic acid (3-HAA) is not a substrate of SibL. In addition, the kynurenine ring is fixed by the hydrogen bonds between its amino and hydroxyl groups and the side chains of Tyr99 (N2), Tyr134 (O3) and Tyr295 (O3). Thus, both cofactor and substrate are poised well for methyl transfer. Curiously, when the active-site pocket was inspected, neither water nor metal ion was found near the C4 position of 3-HK, suggesting that this enzyme may use acid/base-mediated mechanism to catalyze the C-methylation reaction. Indeed, a general base/acid residue is required to initialize the SN_**2**_ reaction in other MT family members^21^. The tyrosine residues in the proximity of active center may be the catalytic base because many other methyltransferases adopt tyrosine as the general base^39^,^40^.

Among the four substrate-binding tyrosine residues, Tyr134 is immediately adjacent to the C4 atom of 3-HK and is most likely to assist in the methyl transfer reaction. The crystal structures of the ternary *Ss-*SibL/SAH/3HK complex and the unbound *Ss-*SibL indicate that: (1) in the ternary complex, the hydroxyl group of Tyr134 makes a direct hydrogen bond to the kynurenine O3 atom; (2) it is directed at the substrate C4 atom with a distance of ~3.3 Å, that is close enough for proton transfer; and (3) upon SAH binding, the entire side chain of Tyr134 flips inversely toward the methylation site from the unbound position, by an approximately 180° rotation. These features suggest that Tyr134 may be deprotonated upon 3-HK binding and subsquently act as a general base in the catalysis. Next to Tyr134, the side chain of Tyr295 is hydrogen bonded to the kynurenine 3-OH group (2.7 Å). It may stabilize the transition state through electron resonance effect of the aromatic ring by temporarily carrying the O3 proton away from 3-HK.

To find out the crucial residues in the general acid/base catalysis, Tyr134 and Tyr295 were replaced with phenylalanine by site-directed mutagenesis. The resulting mutants Y134F and Y295F showed dramatically decreased levels of methyl transferase activity by 98% (Y134F) and 44% (Y295F; Table 2). Consistent with our structure-based mechanism as proposed above, these mutants confirm that the side-chain OH group of Tyr134 is heavily involved in the catalytic reaction, most likely by acting as the principal base. With a high pKa of about 10, Tyr134 does need assistance to be deprotonated before serving the role as a catalytic base. In the apo form structure, the nearby acidic residue Asp132 associates directly with Tyr134. Therefore Asp132 can assist the deprotonation. Upon cofactor binding, the shifted Tyr134 is probably ionized and ready for catalysis. Asp132 is also a strictly conserved amino acid in this class of enzymes (see below).

## Discussion

Although SAM-dependent MTs represent a chemically diverse family of methyl acceptors based on their distinct substrates, most of the known structures belong to class I or III of the recognized families^21^. A DALI search^41^ for the *Ss-*SibL structure returned several known homologous structures (Z-score >20). Most of them belong to the class I O-MT family, despite the low amino acid sequence identity in these hits (<30%). For example, the root-mean-square difference (r.m.s.d.) between the Cα positions of 239 structurally equivalent residues of Ss-SibL and ASMT^38^ is 2.4 Å, and the r.m.s.d. in 214 equivalent Cα positions rises to 3.0 Å for MmcR^42^. Even though these MTs react with various substrates, most of them display the characteristic core SAM-MT fold^17^ (Supplementary Fig. S2). The most highly conserved regions in these homologous proteins are found in their C-terminal domains, such as the glycine-rich motif for SAM binding.

Curiously, only one C-MT, geranyl diphosphate C-methyltransferase (GPPMT), is homologous to *Ss*-SibL structure while displaying a lower score (Z-score = 17.3). The *Ss*-SibL structure shows an r.m.s.d. of 2.4 Å for 162 matched Cα atoms when compared with the GPPMT structure. The main difference between the two proteins is the distinct N-terminal helical regions. GPPMT has a smaller N-terminal region that allows it to be physiologically active as a monomer^34^. Even though both are small-molecule SAM-dependent C-MTs, they vary in size and secondary structure of the N-terminal region. Previous reports also indicated that the MT class I members are evolved from a primordial repertoire of enzymes that typically consist of highly conserved SAM-binding motifs (Supplementary Fig. S3) with diverse substrate-binding residues^37^. Given the presence of the above-described conserved motif, the *Ss*-SibL protein could thus be considered to be a member of this class of enzymes, which act on diverse substrates.

Notably, while the structure of *Ss*-SibL is similar to class I O-MTs, it possesses a unique active site for catalyzing C-transmethylation. For example, the catalytic residues of His255 and Asp256 in ASMT^38^ correspond to two nonpolar Val248 and Ile249 in *Ss*-SibL, suggesting distinct reaction mechanisms with very different active-site configurations in O-MTs and C-MTs. By specific mutagenesis, Tyr134 in the active site of *Ss*-SibL is confirmed to play an important catalytic role during the methyl transfer reaction. Crnovcic *et al.* have characterized AcmI and AcmL^29^. These two enzymes also possess 3-HK methyltransferase activity. Sequence alignment shows that Tyr134 of *Ss*-SibL corresponds well to the highly conserved Tyr138 present in AcmI and AcmL from *Streptomyces anulatus* and Tyr136 in *orf*19 from *Streptomyces refuineus*^29^ (Supplementary Fig. S4), and so is the preceding Asp132. Because the residues involved in substrate binding and catalysis are highly conserved in these C-MTs, they probably share the similar mechanism of methyl transfer in the diverse pathways of antibiotic biosynthesis.

**Figure 5:**
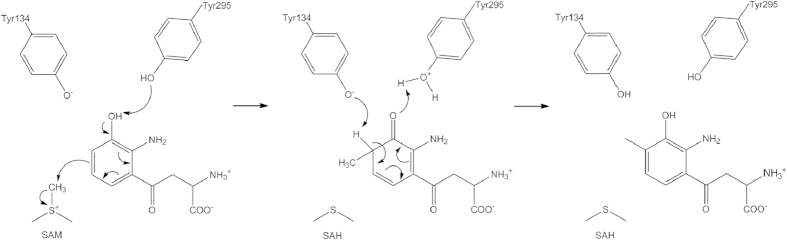
Proposed *Ss*-SibL C-methylation mechanism. Tyr134 and Tyr295 act as a tyrosine clamp to catalyze the transfer of methyl group from SAM to 3-HK.

Most enzymes are well defined by their chemical function and characterized with respect to substrates and products in the BRENDA organization^43^. We searched the archives for *Ss*-SibL but detected very few records. Currently, NPMTs are believed to adopt one of three mechanisms to facilitate methyl transfer via SN_2_ substitution. These mechanisms reveal different reaction features including the distance for the reactive nucleophile to approach the donor or acceptor atoms^15^,^21^. Structures of *Ss*-SibL and its complexes have contributed significantly to the understanding of the substrate discrimination mechanism. Participation of tyrosine residues in catalyzing the acid-base reaction in *Ss*-SibL is thus a novel mechanism for methyl transfer activity, as presented in Fig. 5. Upon SAM binding, the phenolic side chain of Tyr134, which has probably been ionized, flips toward the 3-HK binding site. When the substrate comes in, Tyr134 and Tyr295 form a “tyrosine clamp” that uses the hydroxyl groups to engage the 3-OH group through hydrogen bonding.

The reaction starts with the side chain of Tyr295 orienting its lone pair orbital on the OH group of 3-HK, initially facilitating proton subtraction from the substrate. Subsequent π bond resonance occurs in the aromatic ring, mediating the transfer of electrons from the O3 atom to the C4 atom and resulting in attack on the nearby SAM methyl group. Once the methyl has completely transferred to the C4 position, Tyr134 orients its lone pair toward the C4 hydrogen, performing the second proton subtraction from the substrate. Subsequently, π bond resonance occurs again, that breaks the double bond of the C3 carbonyl group while recovering the proton from Tyr295. During this process, 3-HK is converted to 3-H4MK. In addition, it is noteworthy that the OH group at C3 of 3-HK is much easier to deprotonate due to resonance with the ortho NH_2_ group at C2. This amino group is hydrogen bonded to Tyr99, which is in turn hydrogen bonded to Tyr295. The formation of a proton relay network explains the milder effect of Y295F. Any solvent molecule can serve the role instead. To investigate the role of Tyr99, we also produced the mutant Y99F, but it tended to aggregate and was difficult to purify for activity measurement.

In summary, we have determined the crystal structures of *Ss-*SibL in its unbound form, a binary complex with SAH and a ternary complex with both SAH and 3-HK, which provides a clear picture of the binding pocket. The three structures also reveal an open-to-closed conformational change induced by the cofactor, which is required for formation of the substrate-binding site. In addition, a tyrosine-mediated methyl transfer mechanism is proposed by structural analysis and verified by mutagenesis experiments. The catalytic mechanism for C-MT activity in the *Ss*-SibL protein may apply to other C-MTs in actinomycin or anthramycin biosynthesis.

## Methods

### Cloning, expression, and purification of SibL

The *Ss-*SibL construct was obtained from PCR amplification of *S. sibiricum* genomic DNA as a template and then cloned into a T7 promoter-driven expression system (pET-21b) to enable the expression of a His_6_-tagged recombinant *Ss-*SibL protein in *Escherichia coli* BL21 (DE3) cells. The bacteria were grown at 310 K and 200 rev·min^−1^ in 1 L of LB medium containing 50 mg·l^−1^ ampicillin as a selection agent to an optical density at 600 nm (OD_600_) of 0.6. Expression of *Ss-*SibL was induced by addition of isopropyl β-D-thiogalactopyranoside (IPTG) to a final concentration of 0.5 mM and the culture was grown at 293 K for 16 h. *E. coli* cells from a 4 L culture were harvested by centrifugation at 5,000 × g for 20 min, and the cell pellet was resuspended in buffer A (50 mM Tris-HCl, pH 8.0, 500 mM NaCl, and 5 mM imidazole). Subsequently, the cells were disrupted by sonication and the crude lysate was centrifuged at 20,000 × g for 90 min at 277 K. The clarified supernatant was applied to a Ni-NTA His-bind resin pre-equilibrated with binding buffer. Impurities were removed with Ni-NTA wash buffer (50 mM Tris-HCl, pH 8.0, 500 mM NaCl, and 10 mM imidazole), and the bound *Ss-*SibL was eluted with a 0–200 mM linear gradient of imidazole. Fractions containing *Ss-*SibL were pooled, concentrated by ultrafiltration using an Amicon Ultra-15 3K Centrifugal Filter Device (Millipore; 3-kDa cutoff; Supplementary Fig. S5), and loaded onto a HiLoad 16/60 Superdex-200 size-exclusion column (GE Healthcare) equilibrated with gel filtration buffer (50 mM Tris-HCl, pH 8.0, 100 mM NaCl, 5% glycerol, and 2 mM TCEP). The fractions containing SibL were pooled and concentrated to 10 mg·ml^−1^ for crystallization screening (Supplementary Fig. S5).

### Crystallization and data collection

Initial crystallization trials were performed with commercially available kits (Hampton Research, Emerald BioStructures, and Molecular Dimensions) using the sitting-drop vapor-diffusion method in 24-well VDX plates (Hampton Research), where 1 μl of a protein solution was mixed with an equal amount of the mother liquid. Apo-form crystals were obtained after 2 weeks using a solution containing 50 mM HEPES buffer (pH 7.0), 40% (v/v) tacsimate (pH 7.0), 2 mM spermine, and 2 mM hexamine cobalt (III) chloride. Crystals of *Ss-*SibL were successfully cooled in liquid nitrogen by flash cooling them in the mother liquid supplemented within 20% glycerol as a cryoprotectant for approximately 15 s. X-ray diffraction data were collected at the National Synchrotron Radiation Research Center (NSRRC) BL15A1 in Taiwan.

3-HK as a mixture of d-/l-isomers was purchased from Sigma. Crystals of selenomethionyl-derivatized *Ss-*SibL in complex with SAH and 3-HK were obtained using a solution containing 0.1 M HEPES (pH 7.7), 2.0 M (NH_4_)_2_SO_4_, and 2% PEG400. One SAD dataset was collected at the selenium K-edge from a single crystal at the BL13B1 beamline (NSRRC). Crystals of the native SibL-SAH-3HK and SibL-SAH were grown in a solution consisting of 0.1 M HEPES (pH 7.7), 1.5 M (NH_4_)_2_SO_4_, and 1% PEG400. Diffraction data from the crystal of the ternary and binary complexes were collected at 100 K at beam line BL13C1. All of the diffraction images were indexed and integrated using the HKL2000 processing software^44^. Details of the statistics for the diffraction data are given in Table 1^33^.

### Structure determination

The phenix.autosol and phenix.autobuild wizards were used to solve the structure of the SibL-SAH-3HK complex (a total of 4 of the 8 selenium sites were found) and perform the initial model building. The structures of the apo form and binary complex with SAH were solved using the Phaser program with one monomer of the ternary complex structure as a search model^45^. The structures were completed via multiple manual interactions in the COOT program^46^. Structure refinement was performed using the Phenix.refine program with tight NCS restraints^47^. The quality of all of the models was assessed using the Molprobity program in phenix software^47^. Stereochemical libraries were prepared using phenix.elbow. Coordinates have been deposited in the Protein Data Bank. The accession codes are 4QVG, 4 X 3Q and 4U1Q for the SibL, SibL-SAH and SibL-SAH-3HK structures, respectively.

### Site-directed mutagenesis

*Ss-*SibL mutants were generated with the QuikChange mutagenesis kit (Stratagene) using the parent expression plasmid pET21b-SibL as a template. All of the mutant plasmids were analyzed by complete gene sequencing to ensure that the desired mutations were introduced. All of the mutants were expressed and purified as described for the wild-type enzyme.

### Methyltransferase activity assay

A modified acid-washed charcoal method was used to assess the activity of the recombinant and mutant SibL methyltransferases^48^. The recombinant SibL (20 μM), which was stored at −80 °C, was thawed in an ice bath before the assay. The recombinant SibL was incubated with a reaction mixture (50 mM HEPES, pH 7.0, 50 mM NaCl, 0.1 mM 3-HK, and 1 mM SAM (containing methyl ^3^H-SAM 0.1 μCi 2.0 pmol^−1^, 1 Ci = 3.7 × 10^10^ Bq, PerkinElmer, CT, USA), final volume 100 μL) at 30 °C for 0.5 h. The reactions were terminated by addition of trichloroacetic acid with a final concentration of 2% (v/v). The ^3^H-SAM was precipitated by incubation with 125 μL of acid-washed charcoal mixture (containing 76 mg of charcoal per ml of 0.1 N acetic acid) in an ice bath for 15 min. The supernatant (100 μL), which contained the ^3^H-3H4MK reaction products after centrifugation (17,968 × g at 4 °C for 15 min), was incubated with 3 mL of counting scintillation fluid (PerkinElmer) to measure the amount of radioactivity with a liquid scintillation spectrometer (Pharmacia Co.). Each data point was averaged from triplicate experiments and showed the relative activity in comparison with SibL.

## Author Contributions

P.K.F, S.T.T. and Y.C. designed experiments. S.J.L. and M.C.L. conducted the enzymatic assay. S.C.C. solved the crystal structures. H.C.H., T.P.K. and C.S.Y. analyzed the structures. Y.C. designed, supervised the project and interpreted data. S.C.C., J.S.L., T.P.K. and Y.C. wrote the paper, and all authors have reviewed the manuscript.

## Additional Information

**How to cite this article**: Chen, S.-C. *et al*. Structure and mechanism of an antibiotics-synthesizing 3-hydroxykynurenine C-methyltransferase. *Sci. Rep.* **5**, 10100; doi: 10.1038/srep10100 (2015).

## Supplementary Material

Supplementary Information

## Figures and Tables

**Figure 1:**
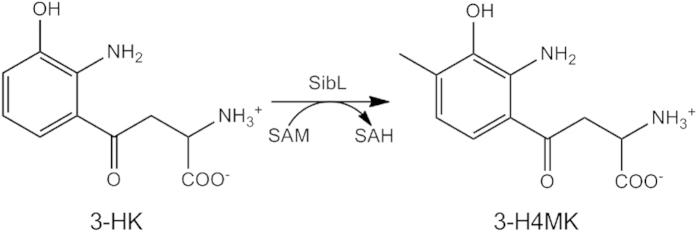
A schematic representation of the Ss-SibL enzymatic reaction.

**Table 1:** X-Ray Data Collection and Refinement Statistics^33^.

**Data collection**	**SibL**	**SibL-SAH**	**SibL-SAH-3HK**
Wavelength (Å)	0.9794	0.9789	0.9762
Space group	*F*222	*P*2_1_2_1_2_1_	*P*2_1_2_1_2_1_
Unit cell (Å)	*a*=102.93	*a*=91.78	*a*=93.30
	*b*=295.42	*b*=112.11	*b*=112.78
	*c*=322.23	*c*=143.70	*c*=143.13
Resolution (Å)	30.0–2.9 (3.0–2.9)	30.0–2.60 (2.69–2.60)	30.0–2.10 (2.18–2.10)
Total observations	324008 (31401)	686246 (67295)	654516 (63471)
Unique reflections	54730 (5414)	46368 (4547)	89602 (8853)
Redundancy	5.9 (5.8)	14.8 (14.8)	7.3 (7.2)
R_merge_ (%)^a^	7.5 (56.0)	7.5 (52.4)	8.1 (49.9)
Completeness (%)	99.7 (100.0)	98.9 (98.5)	99.8 (100.0)
*I/σ* (*I*)	19.3 (3.1)	36.6 (5.0)	23.2 (4.2)

Refinement			
Resolution (Å)	30.0–2.9	30.0–2.6	30.0–2.1
No. of reflections (Working/Free)	52664/2001	46331/3788	89525/2000
R_work_/R_free_ (%)^b^	21.1/26.5	18.3/24.8	17.1/21.3
No. of atoms			
Protein	10569	10660	10724
Ligands			
SAH		104	104
3HK			64
Water		123	995
r.m.s.d.			
Bond lengths (Å)	0.010	0.010	0.008
Bond angles (°)	1.38	1.44	1.15

Ramachandran plot (%)			
Most favored	96.1	97.3	98.0
Allowed	2.9	1.6	1.4

Average B-values (Å^2^)			
Protein	69.7	52.8	36.7
Ligands			
SAH		47.7	31.2
3HK			33.1
Water		48.2	41.6

Values in parentheses show the statistics for the highest resolution shells.

^a^R_merge_ = Σ_hkl_ [(Σ_i_ |I_i_—<I_i_>|)/Σ_i_ |I_i_|]

^b^R_work_ = (Σ_hkl_ ||F_o_|—|F_c_||)/Σ_hkl_ |F_o_|. Rfree was calculated with 2–5% of the data excluded from refinement.

**Table 2:** Measured activity for *Ss*-SibL mutants.

	**Ss-SibL activity (%)**
Wild type	100.0 ± 15.9
Y134F	2.06 ± 1.90
Y295F	56.5 ± 12.7

## References

[b1] Hurley L. H. & Gairola C. Pyrrolo (1,4) benzodiazepine antitumor antibiotics: Biosynthetic studies on the conversion of tryptophan to the anthranilic acid moieties of sibiromycin and tomaymycin. Antimicrob. Agents Chemother. 15, 42–45 (1979). 581831 10.1128/aac.15.1.42 PMC352597

[b2] Hurley L. H. & Speedie M. K. Pyrrolo-(1,4)benzodiazepines antibiotics: Anthramycin, tomaymycin and sibiromycin. Springer, Berlin. 4 (1981). 10.7164/antibiotics.30.349 328469

[b3] Petrusek R. L. *et al.* Pyrrol[1,4]benzodiazepine antibiotics. Proposed structures and characteristics of the in vitro deoxyribonucleic acid adducts of anthramycin, tomaymycin, sibiromycin, and neothramycins A and B. Biochemistry 20, 1111–1119 (1981). 6261786 10.1021/bi00508a011

[b4] Gause G. F. Mechanism of Action of Antimicrobial and Antitumor Agents, Sibiromycin. Antibiotics 3, 269–273 (1975).

[b5] Thurston D. E. *et al.* Effect of A-ring modifications on the DNA-binding behavior and cytotoxicity of pyrrolo[2,1-c][1,4]benzodiazepines. J. Med. Chem. 42, 1951–1964 (1999). 10354403 10.1021/jm981117p

[b6] Kumar R. & Lown J. W. Recent developments in novel pyrrolo[2,1-c][1,4]benzodiazepine conjugates: synthesis and biological evaluation. Mini. Rev. Med. Chem. 3, 323–339 (2003). 12678826 10.2174/1389557033488097

[b7] Giessen T. W., Kraas F. I. & Marahiel M. A. A four-enzyme pathway for 3,5-dihydroxy-4-methylanthranilic acid formation and incorporation into the antitumor antibiotic sibiromycin. Biochemistry 50, 5680–5692 (2011). 21612226 10.1021/bi2006114

[b8] Hurley L. H. Elucidation and Formulation of Novel Biosynthetic Pathways Leading to the Pyrrolo[1,4]Benzodiazepine Antibiotics Anthramycin, Tomaymycin, and Sibiromycin. Acc. Chem. Res. 13, 263–269. (1980).

[b9] Parker K. A. & Babine R. E. Revision of assignment of structure to the pyrrolodiazepinone anti-tumor antibiotic sibiromycin. J. Am. Chem. Soc. 104, 7330–7331. (1982).

[b10] Antonow D. & Thurston D. E. Synthesis of DNA-interactive pyrrolo[2,1-c][1,4]benzodiazepines (PBDs). Chem. Rev. 111, 2815–2864 (2011). 21166464 10.1021/cr100120f

[b11] Cargill C., Bachmann E. & Zbinden G. Effects of daunomycin and anthramycin on electrocardiogram and mitochondrial metabolism of the rat heart. J. Natl. Cancer Inst. 53, 481–486 (1974). 4276468 10.1093/jnci/53.2.481

[b12] Lubawy W. C., Dallam R. A. & Hurley L. H. Protection against anthramycin-induced toxicity in mice by coenzyme Q10. J. Natl. Cancer Inst. 64, 105–109 (1980). 6928034

[b13] Kamal A., Rao M. V., Laxman N., Ramesh G., and Reddy G. S. Recent developments in the design, synthesis and structure activity relationship studies of pyrrolo[2,1-c][1,4]benzodiazepines as DNA-interactive antitumour antibiotics. Curr. Med. Chem.: Anti-Cancer Agents 2, 215–254. (2002). 10.2174/1568011023354119 12678745

[b14] Hurley L. H. Pyrrolo(1,4)benzodiazepine antitumor antibiotics. Comparative aspects of anthramycin, tomaymycin and sibiromycin. J. Antibiot. (Tokyo) 30, 349–370 (1977). 328469 10.7164/antibiotics.30.349

[b15] O’Hagan D. & Schmidberger J. W. Enzymes that catalyse SN2 reaction mechanisms. Nat. Prod. Rep. 27, 900–918, doi: 10.1039/b919371p (2010). 20372740

[b16] Luka Z., Mudd S. H. & Wagner C. Glycine N-methyltransferase and regulation of S-adenosylmethionine levels. J. Biol. Chem. 284, 22507–22511, doi: 10.1074/jbc.R109.019273 (2009). 19483083 PMC2755656

[b17] Martin J. L. & McMillan F. M. SAM (dependent) I AM: the S-adenosylmethionine-dependent methyltransferase fold. Curr. Opin. Struct. Biol. 12, 783–793 (2002). 12504684 10.1016/s0959-440x(02)00391-3

[b18] Schubert H. L., Blumenthal R. M. & Cheng X. Many paths to methyltransfer: a chronicle of convergence. Trends Biochem. Sci. 28, 329–335, doi: 10.1016/S0968-0004(03)00090-2 (2003). 12826405 PMC2758044

[b19] Wlodarski T. *et al.* Comprehensive structural and substrate specificity classification of the Saccharomyces cerevisiae methyltransferome. PLoS One 6, e23168, doi: 10.1371/journal.pone.0023168 (2011). 21858014 PMC3153492

[b20] Barakat A. *et al.* Comparative genomics and evolutionary analyses of the O-methyltransferase gene family in Populus. Gene 479, 37–46 (2011). 21338660 10.1016/j.gene.2011.02.008

[b21] Liscombe D. K., Louie G. V. & Noel J. P. Architectures, mechanisms and molecular evolution of natural product methyltransferases. Nat. Prod. Rep. 29, 1238–1250 (2012). 22850796 10.1039/c2np20029e

[b22] Liscombe D. K., Usera A. R. & O’Connor S. E. Homolog of tocopherol C methyltransferases catalyzes N methylation in anticancer alkaloid biosynthesis. Proc. Natl. Acad. Sci. USA 107, 18793–18798 (2010). 20956330 10.1073/pnas.1009003107 PMC2973921

[b23] Zhao N. *et al.* Structural, biochemical, and phylogenetic analyses suggest that indole-3-acetic acid methyltransferase is an evolutionarily ancient member of the SABATH family. Plant Physiol. 146, 455–467 (2008). 18162595 10.1104/pp.107.110049 PMC2245846

[b24] Jansson A., Koskiniemi H., Mantsala P., Niemi J. & Schneider G. Crystal structure of a ternary complex of DnrK, a methyltransferase in daunorubicin biosynthesis, with bound products. J. Biol. Chem. 279, 41149–41156 (2004). 15273252 10.1074/jbc.M407081200

[b25] Gomez Garcia I. *et al.* The crystal structure of the novobiocin biosynthetic enzyme NovP: the first representative structure for the TylF O-methyltransferase superfamily. J. Mol. Biol. 395, 390–407, doi: 10.1016/j.jmb.2009.10.045 (2010). 19857499 PMC2813333

[b26] Akey D. L. *et al.* A new structural form in the SAM/metal-dependent omethyltransferase family: MycE from the mycinamicin biosynthetic pathway. J. Mol. Biol. 413, 438–450, doi: 10.1016/j.jmb.2011.08.040 (2011). 21884704 PMC3193595

[b27] Kelley L. A. & Sternberg M. J. Protein structure prediction on the Web: a case study using the Phyre server. Nat. Protoc. 4, 363–371, doi: 10.1038/nprot.2009.2 (2009). 19247286

[b28] Li W., Khullar A., Chou S., Sacramo A. & Gerratana B. Biosynthesis of sibiromycin, a potent antitumor antibiotic. Appl. Environ. Microbiol. 75, 2869–2878 (2009). 19270142 10.1128/AEM.02326-08 PMC2681668

[b29] Crnovcic I., Sussmuth R. & Keller U. Aromatic C-methyltransferases with antipodal stereoselectivity for structurally diverse phenolic amino acids catalyze the methylation step in the biosynthesis of the actinomycin chromophore. Biochemistry 49, 9698–9705, doi: 10.1021/bi101422r (2010). 20945860

[b30] Zubieta C., He X. Z., Dixon R. A. & Noel J. P. Structures of two natural product methyltransferases reveal the basis for substrate specificity in plant O-methyltransferases. Nat. Struct. Biol. 8, 271–279 (2001). 11224575 10.1038/85029

[b31] Cooke H. A., Guenther E. L., Luo Y., Shen B. & Bruner S. D. Molecular basis of substrate promiscuity for the SAM-dependent O-methyltransferase NcsB1, involved in the biosynthesis of the enediyne antitumor antibiotic neocarzinostatin. Biochemistry 48, 9590–9598 (2009). 19702337 10.1021/bi901257q PMC4500170

[b32] Rossmann M. G., Moras D. & Olsen K. W. Chemical and biological evolution of nucleotide-binding protein. Nature 250, 194–199 (1974). 4368490 10.1038/250194a0

[b33] Fu P. K. *et al.* Crystallization and preliminary X-ray diffraction analysis of SibL, a SAM-dependent C-methyltransferase from Streptosporangium sibiricum. Int. J. Multidiscip. Des. Dev. 1, 19–23 (2014).

[b34] Koksal M., Chou W. K., Cane D. E. & Christianson D. W. Structure of geranyl diphosphate C-methyltransferase from Streptomyces coelicolor and implications for the mechanism of isoprenoid modification. Biochemistry 51, 3003–3010 (2012). 22455498 10.1021/bi300109c PMC3323675

[b35] Hoffman J. L. Chromatographic analysis of the chiral and covalent instability of S-adenosyl-L-methionine. Biochemistry 25, 4444–4449 (1986). 3530324 10.1021/bi00363a041

[b36] Taylor D., Cawley G. & Hayward S. Quantitative method for the assignment of hinge and shear mechanism in protein domain movements. Bioinformatics 30, 3189–3196, doi: 10.1093/bioinformatics/btu506 (2014). 25078396 PMC4221117

[b37] Fauman E. B., Blumenthal R. M. & Cheng X. Structure and evolution of AdoMet-dependent methyltransferases. Singapore, World Scientific Inc., 1–38, doi: 10.1142/9789812813077_0001 (1999).

[b38] Botros H. G. *et al.* Crystal structure and functional mapping of human ASMT, the last enzyme of the melatonin synthesis pathway. J. Pineal. Res. 54, 46–57 (2013). 22775292 10.1111/j.1600-079X.2012.01020.x

[b39] Huang C. C., Smith C. V., Glickman M. S., Jacobs W. R. Jr. & Sacchettini J. C. Crystal structures of mycolic acid cyclopropane synthases from Mycobacterium tuberculosis. J. Biol. Chem. 277, 11559–11569, doi: 10.1074/jbc.M111698200 (2002). 11756461

[b40] Takata Y. *et al.* Catalytic mechanism of glycine N-methyltransferase. Biochemistry 42, 8394–8402, doi: 10.1021/bi034245a (2003). 12859184

[b41] Holm L. & Rosenstrom P. Dali server: conservation mapping in 3D. Nucleic Acids Res. 38, W545–549, doi: 10.1093/nar/gkq366 (2010). 20457744 PMC2896194

[b42] Singh S. *et al.* Structural characterization of the mitomycin 7-O-methyltransferase. Proteins 79, 2181–2188, doi: 10.1002/prot.23040 (2011). 21538548 PMC3115387

[b43] Schomburg I. *et al.* BRENDA in 2013: integrated reactions, kinetic data, enzyme function data, improved disease classification: new options and contents in BRENDA. Nucleic. Acids. Res. 41, D764–772 (2013). 23203881 10.1093/nar/gks1049 PMC3531171

[b44] Otwinowski Z. & Minor W. Processing of X-ray Diffraction Data Collected in Oscillation Mode Methods Enzymol. 276, 307–326, (1997). 10.1016/S0076-6879(97)76066-X 27754618

[b45] McCoy A. J. *et al.* Phaser crystallographic software. J. Appl. Crystallogr. 40, 658–674 (2007). 19461840 10.1107/S0021889807021206 PMC2483472

[b46] Emsley P., Lohkamp B., Scott W. G. & Cowtan K. Features and development of Coot. Acta. Crystallogr. D. Biol. Crystallogr. 66, 486–501 (2010). 20383002 10.1107/S0907444910007493 PMC2852313

[b47] Afonine P. V. *et al.* Towards automated crystallographic structure refinement with phenix.refine. Acta. Crystallogr. D. Biol. Crystallogr. 68, 352–367 (2012). 22505256 10.1107/S0907444912001308 PMC3322595

[b48] Cook R. J. & Wagner C. Glycine N-methyltransferase is a folate binding protein of rat liver cytosol. Proc. Natl. Acad. Sci. USA 81, 3631–3634 (1984). 6587377 10.1073/pnas.81.12.3631 PMC345272

