# Supplementary material for: Structure and mechanism of an antibiotics-synthesizing 3-hydroxykynurenine C-methyltransferase
Source: Sci Rep. 2015 May 11;5:10100. doi: 10.1038/srep10100 (PMC4426599; doi:10.1038/srep10100)
Supplement: Supplementary Information [file srep10100-s1.pdf]

Title

Structure and mechanism of an antibiotics-synthesizing 3-hydroxykynurenine C-methyltransferase

Author list

Sheng-Chia Chen, Chi-Hung Huang, Shu-Jung Lai, Jai-Shin Liu, Pin-Kuei Fu, Shih-Ting Tseng, Chia Shin Yang, Mei-Chin Lai, Tzu-Ping Ko, and Yeh Chen

Supplementary Figure Legends

Supplementary Figure S1. The sibiromycin biosynthesis pathway. The last common precursor in the anthramycin biosynthesis is indicated by additional arrows.

Supplementary Figure S2. Superposition of the Ss-SibL (green), ASMT (magenta) and MmcR(cyan).

Supplementary Figure S3. Structural and sequence alignments of representative methyltransferases. (1) Ss-SibL. (2) ASMT: human N-acetylserotonin MT. (3) MmcR: mitomycin 7-O-MT. (4) NcsB1: SAM-dependent O-MT from *Streptomyces carzinostaticus*.

Supplementary Figure S4. Structure-based amino acid sequence alignment. The sequences include *Ss*-SibL, AcmL from *Streptomyces anulatus*, AcmI from *Streptomyces anulatus* and ORF19 from *Streptomyces refuineus.* Secondary structural elements are plotted above the sequences. The β-strands (β1–β9) and α-helices (α1–α15) are numbered from the N-terminus. TT, β-turns; η (η1–η5), 3_{10} helix. Identical and similar residues are boxed in red and white, respectively. The SAH-interacting

residues are indicated in triangle blue. Red circles highlight the residues involved in substrate binding.

Supplementary Figure S5. SDS-PAGE of purified *Ss*-SibL. Lane M, molecular-weight markers in kDa; lane 1, *Ss*-SibL purified by Ni-column; lane 2, *Ss*-SibL purified by gel filtration.

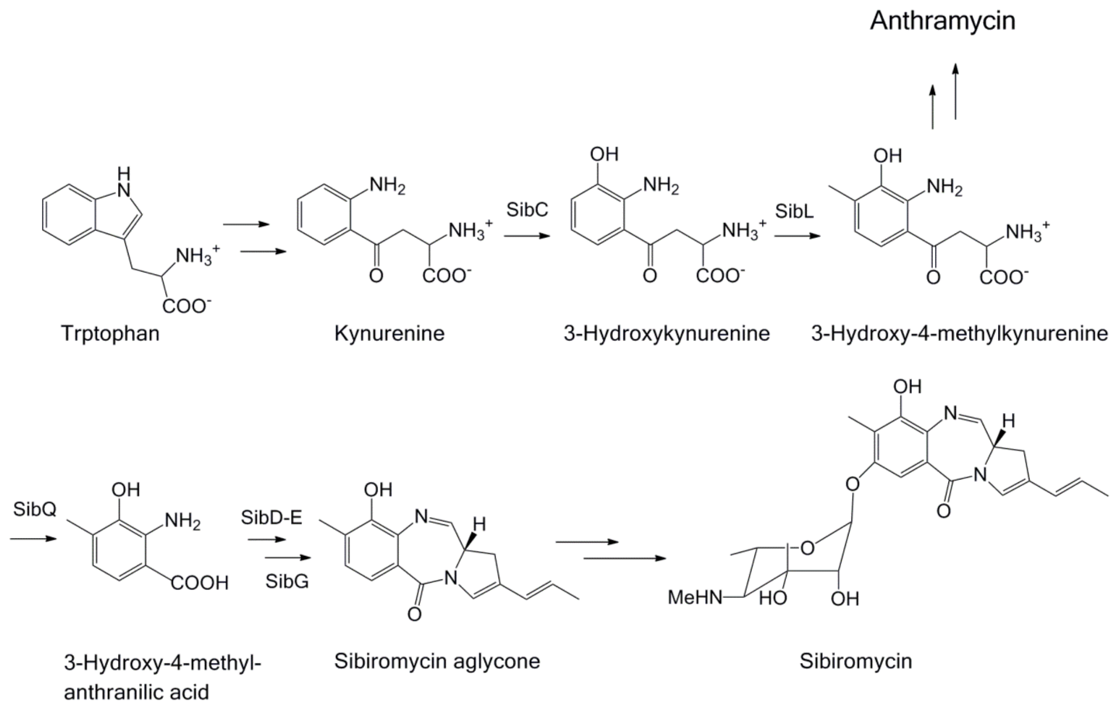

Supplementary Figure S1

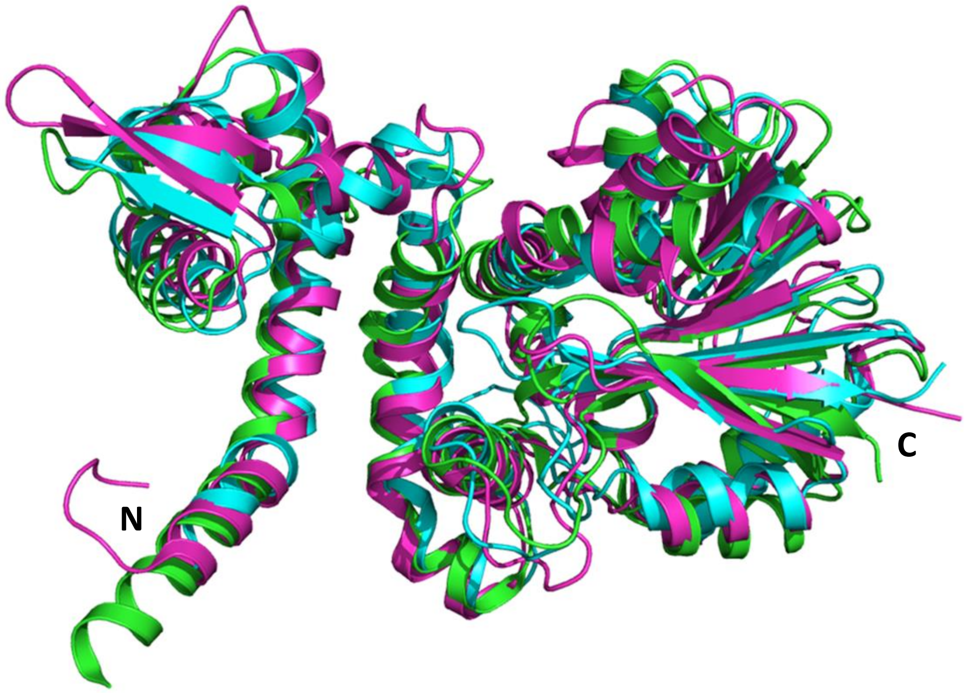

Supplementary Figure S2.

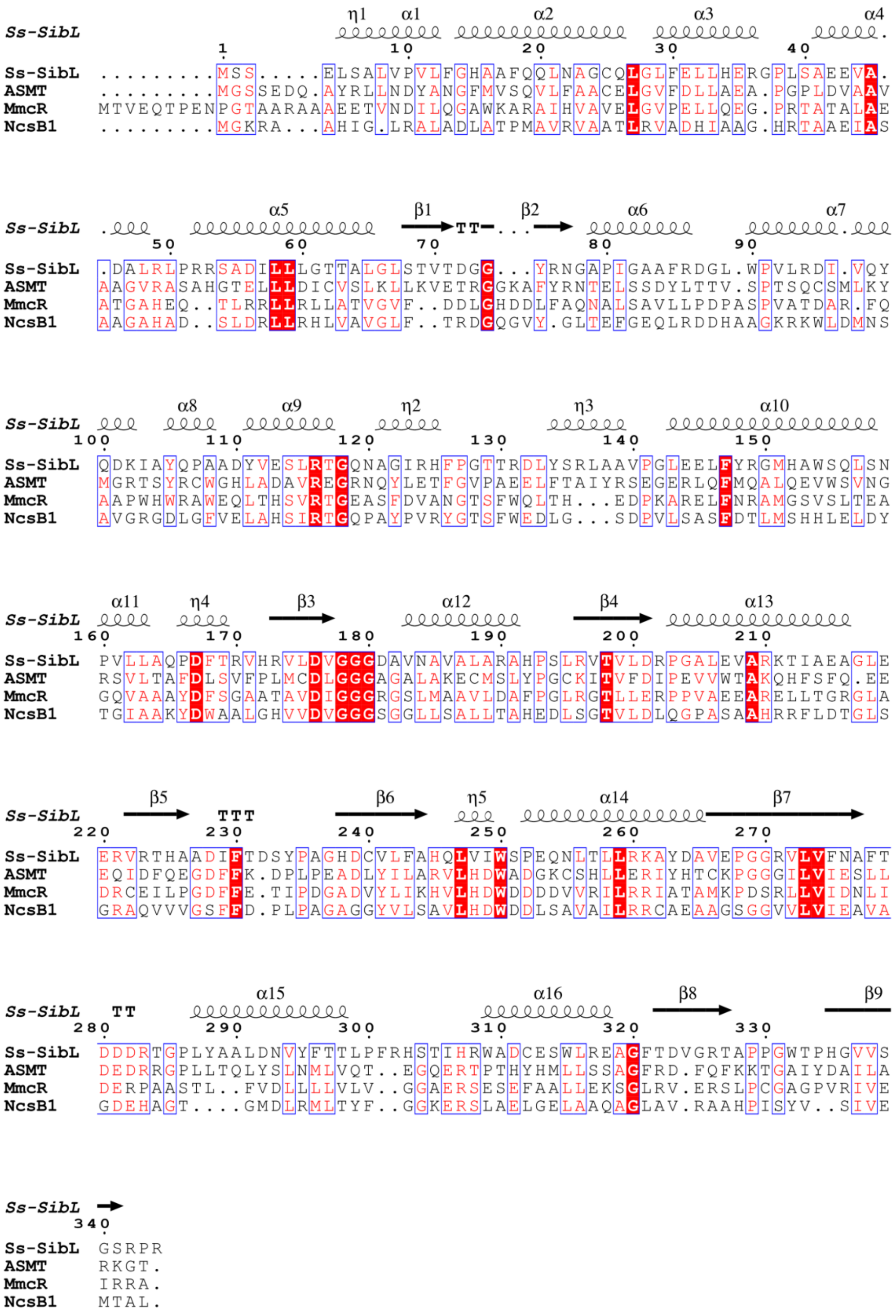

Supplementary Figure S3.

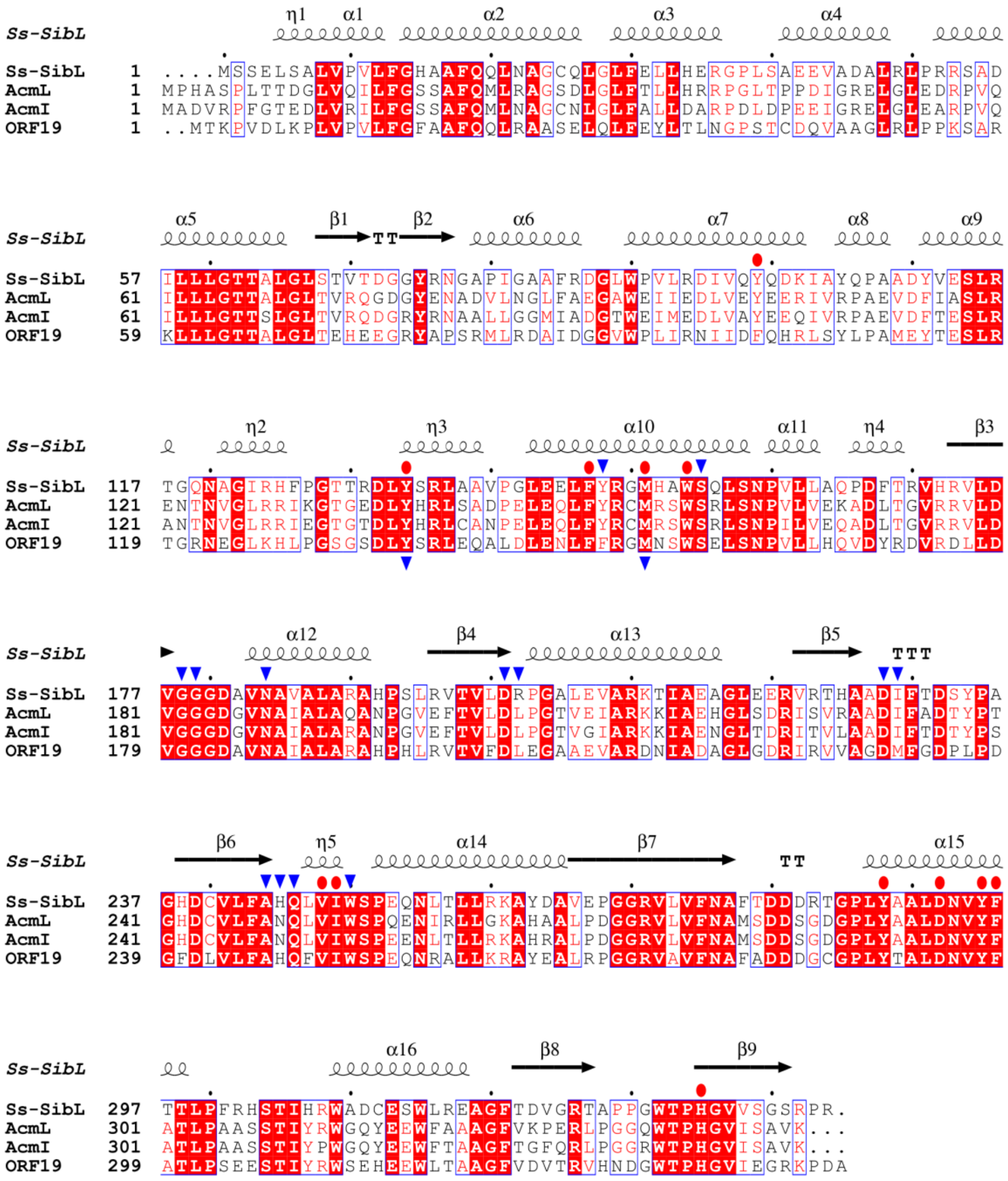

Supplementary Figure S4.

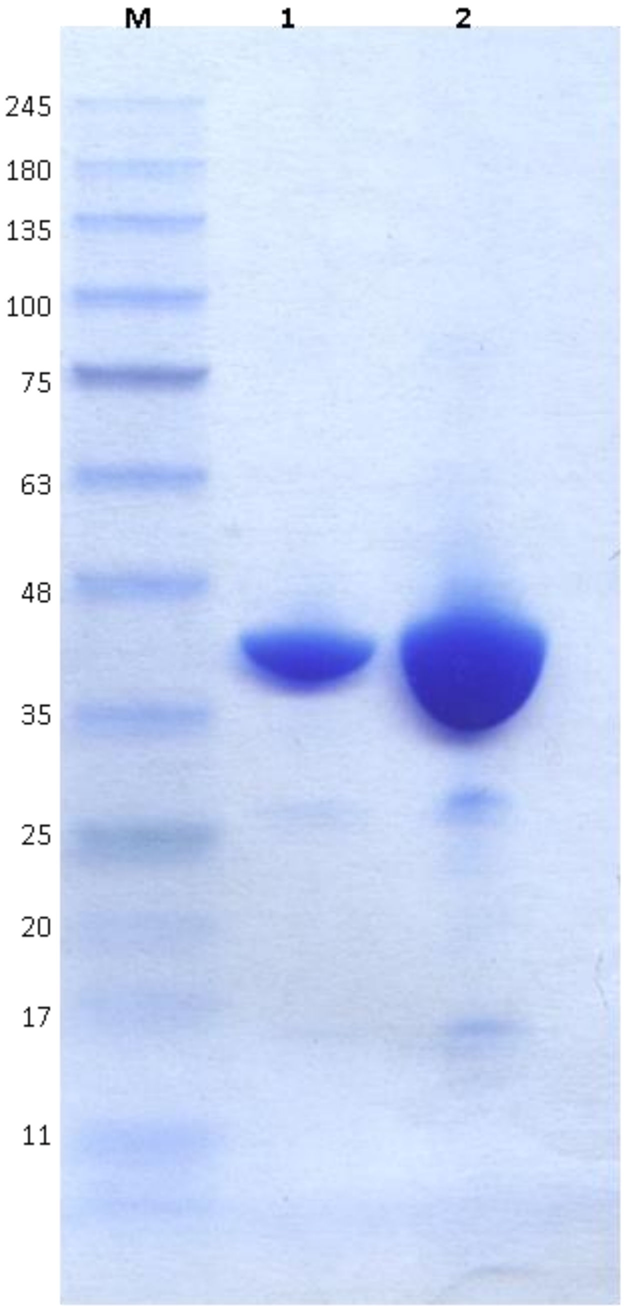

Supplementary Figure S5.
